# Adverse wind conditions during northward Sahara crossings increase the in‐flight mortality of Black‐tailed Godwits

**DOI:** 10.1111/ele.13387

**Published:** 2019-09-17

**Authors:** A. H. Jelle Loonstra, Mo A. Verhoeven, Nathan R. Senner, Christiaan Both, Theunis Piersma

**Affiliations:** ^1^ Conservation Ecology Group Groningen Institute for Evolutionary Life Sciences (GELIFES) University of Groningen P.O. Box 11103 9700 CC Groningen The Netherlands; ^2^ Department of Biological Sciences University of South Carolina 715 Sumter Street Columbia SC 29208 USA; ^3^ NIOZ Royal Netherlands Institute for Sea Research Department of Coastal Systems and Utrecht University P.O. Box 59 1790 AB Den Burg Texel The Netherlands

**Keywords:** *Limosa limosa limosa*, migration, mortality, Sahara desert, wind assistance

## Abstract

Long‐distance migratory flights are predicted to be associated with higher mortality rates when individuals encounter adverse weather conditions. However, directly connecting environmental conditions experienced in‐flight with the survival of migrants has proven difficult. We studied how the in‐flight mortality of 53 satellite‐tagged Black‐tailed Godwits (*Limosa limosa limosa*) during 132 crossings of the Sahara Desert, a major geographical barrier along their migration route between The Netherlands and sub‐Saharan Africa, is correlated with the experienced wind conditions and departure date during both southward and northward migration. We show that godwits experienced higher wind assistance during southward crossings, which seems to reflect local prevailing trade winds. Critically, we found that fatal northward crossings (15 deaths during 61 crossings) were associated with adverse wind conditions. Wind conditions during migration can thus directly influence vital rates. Changing wind conditions associated with global change may thus profoundly influence the costs of long‐distance migration in the future.

## Graphical abstract

Black‐tailed Godwits (Limosa limosa limosa) cross the Sahara Desert twice a year while migrating from their breeding grounds to the sub‐Saharan African wintering grounds. During southward Sahara crossings, godwits experience a higher wind assistance than during northward crossings. Critically, fatal northward crossings were associated with a low wind assistance. In‐flight experienced wind conditions can thus influence the probability to successfully cross a major geographic barrier. Photo: Jan van de Kam.

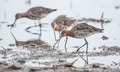

## Introduction

The migration of birds travelling between wintering and breeding areas is widespread and considered to be an adaptive response to fluctuating seasonal environments (Alerstam *et al.* 2013; Winger *et al.* 2019). Despite its presumed adaptiveness, considerable variation in migratory behaviour exists both within and among individuals, and across populations (Newton 2008). To explain why this variation exists, it is necessary to understand how it affects the reproductive performance and survival of individuals, as well as whether it is the result of ‘pre‐functional’ differences among individuals (Hogan 2017) or the result of different developmental trajectories (Piersma 2011). The large‐scale nature of migration has made developing this understanding difficult, especially when determining the mortality rates associated with different migratory strategies (Shamoun‐Baranes *et al.* 2017). However, with the advent of miniaturised tracking devices and advanced survival analyses, we are now better able to study these associations *in situ* (Sillett & Holmes 2002; Strandberg *et al.* 2010; Klaassen *et al.* 2014; Lok *et al.* 2015; Rockwell *et al.* 2016; Ward *et al.* 2018; Senner *et al.* 2019). Although most of these studies have found evidence consistent with long‐standing predictions that migration is the most hazardous part of the migratory annual cycle, examples of other portions of the annual cycle exhibiting higher mortality rates also exist (Leyrer *et al.* 2013). This raises questions about possible population‐specific selective pressures during migration and how populations are able to adapt to these pressures (Rakhimberdiev *et al.* 2015).

Although the quantification of mortality during migration provides essential information about the costs of migration, to directly infer selection pressures, we need to know the proximate cause(s) of these mortality events (Alerstam *et al.* 2013). Previous studies have hypothesised that numerous factors could lead to elevated mortality rates during migration, including the use of unfamiliar habitats and stopover sites, the high energetic costs of migration and the occurrence of inclement weather conditions, such as strong head winds, sandstorms and extreme rainfall (Newton 2007). Among these, and especially for migrations crossing large inhospitable geographical features that lack emergency stopover sites – like deserts and oceans – the amount and predictability of wind assistance during migration are thought to affect the success of migratory flights the most (Erni *et al.* 2005; Shamoun‐Baranes *et al.* 2010; Aurbach *et al.* 2018; Ward *et al.* 2018).

Continental Black‐tailed Godwits (*Limosa limosa limosa*; hereafter: godwits) breed primarily in The Netherlands, with approximately 75% of the population crossing the Sahara Desert twice each year to winter in sub‐Saharan Africa, whereas the remaining 25% stay to winter on the Iberian Peninsula (Márquez‐Ferrando *et al.* 2014; Kentie *et al.* 2017). Previous work on the annual survival of godwits has revealed that northward Sahara crossings are associated with the highest mortality risk of any portion of the annual cycle while southward crossings are less dangerous (Senner *et al.* 2019). Here, we evaluate whether individual godwits tracked with satellite transmitters experience seasonal differences in the amount of wind assistance that they encounter during southward and northward Sahara crossings. In addition, we also determine whether the increased mortality risk during northward Sahara crossings is correlated with an increase in wind‐induced flight costs or departure date. Given the prevailing north‐easterly trade winds across the Sahara (Hayward & Oguntoyinbo 1987; Piersma & van de Sant 1992; Evan *et al.* 2016), we hypothesise that during southward Sahara crossings godwits are more likely to experience better wind conditions than during northward Sahara crossings. Although these favourable winds occur at lower altitudes during southward migration – altitudes which coincide with higher temperatures that could force godwits to experience hyperthermia and dehydration (Liechti & Schmaljohann 2007; Schmaljohann *et al.* 2008; Senner *et al.* 2018) – our previous work (Senner *et al.* 2019) has shown that southward crossings are not associated with elevated mortality. We therefore predict that the elevated in‐flight mortalities during northward Sahara crossings are related to more adverse wind conditions.

## Materials and Methods

### Data collection

Migratory tracks of godwits crossing the Sahara were extracted from 49 adult godwits equipped with PTT‐100 9.5 g solar satellite transmitters (duty cycle: 8‐h transmission, 24‐h charge (*n = *32) or 10‐h transmission, 48‐h charge (*n = *15); Microwave Telemetry, Inc.) and four adult godwits equipped with PTT‐100 5 g solar satellite transmitters (duty cycle: 8‐h transmission, 24‐h charge; Microwave Telemetry, Inc.). Transmitters were deployed from 2013 to 2015 at staging areas in southern Spain and Portugal and from 2015 to 2017 on the Dutch breeding grounds. We successfully documented 71 southward Sahara crossings from 2013 to 2018 and 61 northward Sahara crossings from 2014 to 2018 (Table 1). All transmitters were attached with a leg‐loop harness; see Senner *et al. *(2015) for details on capture and attachment methods. Senner *et al. *(2019) documented a lower *annual* survival probability for godwits outfitted with 9.5 g satellite transmitters than those carrying other tracking devices or only colour rings. However, because of the seasonal discrepancy in survival during migratory flights of godwits outfitted with satellite transmitters across the Sahara (e.g. high survival rate during southward Sahara crossings, but low during northward crossings; Senner *et al.* 2019), we argue that carrying a tag itself does not explain the interindividual variation in mortality during migration across the Sahara.

**Table 1:** Sample size of the Sahara crossings used in this study

Year	Total number of southward Sahara crossings (died)	Total number of northward Sahara crossings (died)
2013	5 (0)	‐
2014	10 (0)	6 (3)
2015	24 (0)	6 (1)
2016	17 (0)	23 (4)
2017	8 (0)	17 (5)
2018	7 (0)	9 (2)
Total	71 (0)	61 (15)

Overview of the number of northward and southward Sahara crossings by godwits per year used in this study, whereby the number between the brackets indicates how many of the total died.

Locations were retrieved and extracted from the CLS tracking system (://www.argos-system.org) and passed through the Hybrid filter (DAF) algorithm (Douglas *et al.* 2012). We retained locations with qualities of 3, 2, 1, 0, A and B with, on average, 8 ± 1 SD locations per individual duty cycle and 7 ± 2 SD locations per individual during a trans‐Saharan flight. To determine the fate of birds during a Sahara crossing, we used three different diagnostic rules: (1) birds outfitted with a 9.5 g transmitter were considered dead when the activity sensor of their tag remained constant, (2) birds outfitted with a 5 g transmitter were considered dead when the temperature sensor started to follow a diurnal rhythm and (3) birds outfitted with either a 5 g or 9.5 g transmitter were considered dead when their transmitter stopped transmitting within a migratory bout and never turned on again. In addition to the deployment of satellite transmitters, and to ensure that the sudden loss of a bird was not the result of a malfunctioning transmitter, we also ringed each bird with a unique combination of four colour rings and a colour flag. We then subsequently attempted to resight these colour‐marked individuals throughout their annual cycle (see: Loonstra *et al.* 2019); with an annual resighting probability of 0.92 for tagged birds that were alive, no individual whose transmitter ceased functioning during a trans‐Saharan flight was subsequently resighted.

### Simulated wind assistance

PTT‐100 solar transmitters provide unique information on in‐flight mortality; however, their limited duty cycles hamper documenting an individual’s entire migratory route and altitude during migration. As a result, we cannot directly infer the wind assistance from an observed Sahara crossing nor the exact location of a mortality event. Hence, we have to simulate the minimal flight duration (e.g. flight costs) of a migratory flight resulting from the wind conditions an individual experienced during a Sahara crossing.

**Figure 1:**
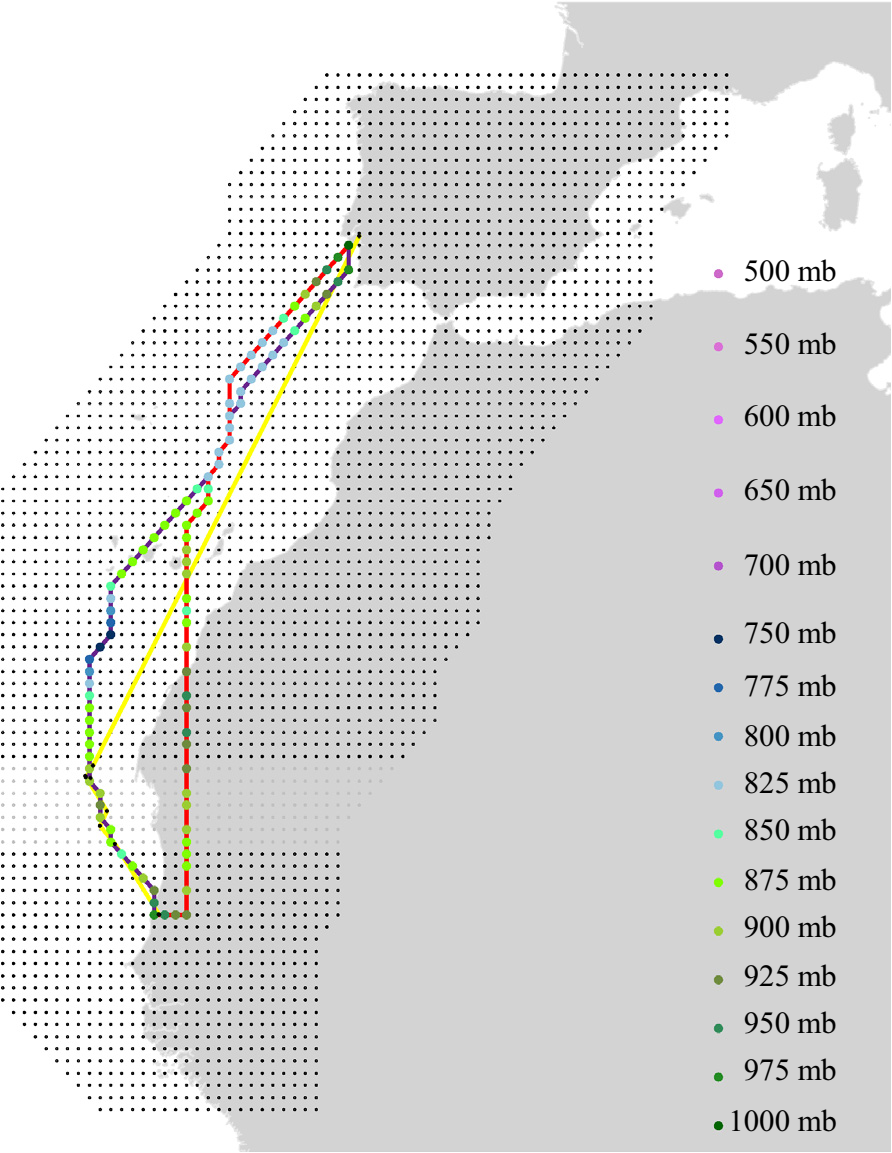
Study area overlain with the grid of nodes that served as the area of the simulations. Geographical overview of the nodes: grey and dark grey points (uninformed grid) and dark grey points (informed grid) separated by 0.4°. All nodes also had a vertical component (1000 mb–500 mb). To clarify the results, we display the route and altitude of a simulated northward migration within an informed grid (purple line) and uninformed grid (red line) for an individual that migrated from the Djoudj, Senegal to the Tagus Estuary, Portugal (yellow line and black points). The weight and connection between nodes are visually explained in Figure S1.

These simulations were performed by calculating the flight duration of the simulated optimal route with respect to the wind support obtained from a three‐dimensional map of connected nodes (Dijkstra 1959; Kranstauber *et al.* 2015; Figs 1 and S1). Because empirical tracking data showed that godwits only use a ‘narrow’ corridor when crossing the Sahara, we restricted the locations of our nodes to the migratory zone of godwits (Fig. 1). The spatial resolution of the nodes was 0.4 degrees by 0.4 degrees, and within the grid we allowed horizontal, upward and downward movements between nodes (see: Fig. S1). With minimal flight altitudes reaching ground level and maximal measured flight altitudes of 5956 m (Senner *et al.* 2018), we included 16 different pressure layers (1000, 975, 950, 925, 900, 875, 850, 825, 800, 775, 750, 700, 650, 600, 550 and 500 millibar) and thus cover the entire altitudinal range of godwits during migration. The weight of a connection between two nodes was described as the travel time between those two nodes. The travel time was calculated with the function ‘NCEP.tailwind’ in the R package ‘RNCEP’ assuming a constant airspeed of 18.05 m/s (Kemp *et al.* 2012; Senner *et al.* 2018) and depended on the distance, direction and groundspeed between two nodes. The wind conditions at a node were matched with the time‐wise nearest ECMWF‐Interim wind data (0:00, 6:00, 12:00, 18:00) and the estimated arrival time at a node, which was based on the departure time from the departing node and the flight time of the straight line between the departing node and node of interest (Dee *et al.* 2011). To parameterise each simulation, we determined the departure and arrival node, as well as departure time, and constrained the movements in the grid according to the retrieved in‐flight locations from each individual migration (Fig. 1; blue line).

For birds that died during migration, we were not able to determine the arrival node; in these cases, we used the arrival node of a previous northward migration, as adult godwits repeatedly use the same staging location on the Iberian Peninsula (*r* = 0.93; Verhoeven *et al.* 2018). In all of our simulations, we assumed that a bird uses flapping flight, minimises the total time during migration (Hedenström *et al.* 2008; Senner *et al.* 2018), and is able to predict and anticipate future wind conditions at departure and during flight (Gill *et al.* 2014). In this framework, the flight time of the optimal simulated route thus increases when an individual faced stronger headwinds. The minimal flight time can thus be considered an indirect measure of the minimal wind‐induced total flight cost (Pennycuick 2008).

### Simulation robustness

Within our simulation framework, we assumed that godwits minimise their flight time by anticipating future wind conditions. To assess the robustness of this assumption, we performed a second, ‘uninformed’ simulation in which we did not constrain the grid of nodes on the basis of the retrieved in‐flight locations (Fig. 1; red line). The flight duration of these ‘uninformed’ simulated routes was thus the time‐wise most efficient route from a departing node to an arriving node at a departure moment and, per definition, of equal or shorter duration than the flight time of the ‘informed’ simulation or actual route (Fig. 1; blue line). The difference between the ‘informed’ and ‘uninformed’ simulations then served as a measure of our time‐minimisation assumption; to infer whether our assumption was violated by a specific group (dying vs. surviving or northward vs. southward Sahara crossing), we also compared the differences between these groups.

### Statistical analysis

We used the simulated individual minimal flight time as a response variable in a linear mixed effect model in the R package ‘lme4’ (Bates *et al.* 2015) to infer whether minimal flight time during southward migration significantly differed from that during northward migration. To account for the non‐independency of migrations from the same individual, we included individual as a random intercept.

Second, we used a generalised linear mixed effect model with a binomial error distribution to compare the optimal simulated flight duration between godwits that successfully crossed the Sahara and those that died during northward migration. To test whether the departure date from Africa during northward migration influenced the in‐flight survival probability, we included the difference between individual departure date and the 5‐year mean (2014–2018) departure date from Africa (hereafter relative departure date). Because of the use of different types of transmitters (i.e. 5 g and 9.5 g), we also included transmitter type as a fixed effect; to account for the non‐independency of migrations from the same individual, we included individual as random intercept. Lastly, to compare the flight time of the informed and uninformed simulations, we used a linear mixed effect model with individual as random intercept.

The statistical significance of the fixed effects in all models was assessed using parametric bootstrapping tests using the ‘pbkrtest’ package (Halekoh & Højsgaard 2014). All statistical analyses were performed using R (v. 3.4.3; R Core Development Team 2018).

## Results

**Figure 2:**
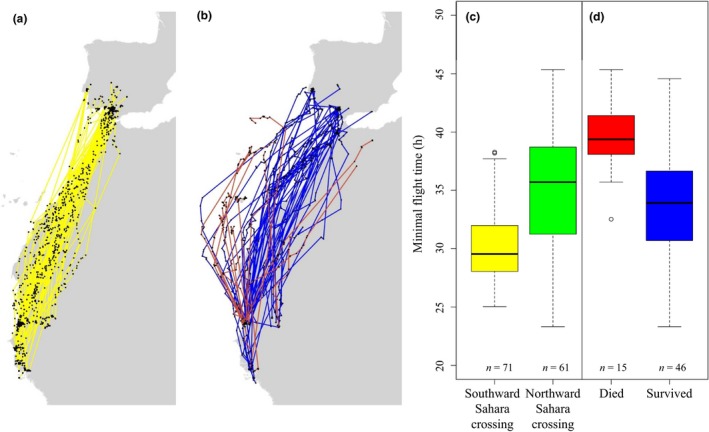
Migratory routes and minimal flight time for godwits crossing the Sahara. (a) Observed migratory routes of godwits during southward (yellow lines) migration; (b) observed migratory routes of godwits during northward migration, red lines for unsuccessful crossings and blue for successful crossings; (c) minimal flight times during southward (yellow) and all northward (green) Sahara crossings; (d) minimal flight times for godwits that either successfully (blue) or unsuccessfully (red) crossed the Sahara during northward migration.

We determined the fate and minimal flight time of 71 crossings of the Sahara during southward migration and 61 during northward migration for a total of 53 unique godwits (50 females and 3 males). None of the birds in our dataset died during a southward Sahara crossing, whereas 15 died during a northward crossing (Table 1, Fig. 2a and b).

The average minimal flight time of godwits during southward Sahara crossings was 30.24 ± 3.01 h (mean ± SD). This was significantly shorter than the average minimal (35.18 ± 5.10 h (mean ± SD)) flight time during northward Sahara crossings (*P *< 0.001; Fig. 2c). Mortality during northward Sahara crossings was neither influenced by tag type (*P = *0.77) nor by departure date (*P* = 0.52). However, for godwits that successfully crossed the Sahara during northward migration, the minimal flight time was on average more than 6 h shorter (33.70 ± 4.72 h (mean ± SD)) than for birds that died (39.72 ± 3.25 h (mean ± SD); *P* < 0.01; Fig. 2d).

The minimal simulated flight time of the uninformed simulations was on average 3.4% shorter than an informed simulation (*P *< 0.05). This difference, however, was not significantly larger between northward and southward Sahara crossings (*P* = 0.56) and did not differ between birds that successfully crossed the Sahara during northward migration and birds that did not (*P* = 0.25).

## Discussion

Wind conditions during migration have been suggested to be one of the strongest factors moulding the migratory routes and performance of migratory birds (Alerstam 1979; Kranstauber *et al.* 2015). We show that during southward Sahara crossings, godwits experience a higher wind assistance (i.e. resulting in shorter flight times) than during northward crossings, which is most likely the result of prevailing trade winds blowing from a north‐easterly direction (Hayward & Oguntoyinbo 1987; Piersma & van de Sant 1992; Evan *et al.* 2016). Furthermore, we show that during northward Sahara crossings, godwits not only migrated in less favourable wind conditions but that flights with longer minimal flight times (i.e. lower wind assistance) were associated with an increased in‐flight mortality risk.

### Estimating wind assistance during migration

Previous studies (Shamoun‐Baranes *et al.* 2010; Gill *et al.* 2014; Kranstauber *et al.* 2015) have provided different methods to estimate the amount of wind assistance experienced by individual birds during migration. While these methods can be applied within a 2D framework (Kranstauber *et al.* 2015) or along a fixed route (Shamoun‐Baranes *et al.* 2010; Gill *et al.* 2014), we suggest that our implementation of a 3D grid greatly improves the estimation of wind assistance during migration. This follows because godwits – and presumably other long‐distance migratory birds as well – have been shown to frequently change their altitude during migratory flights (Senner *et al.* 2018). However, our method, like previous methods, represents an underestimation of the true experienced flight times, as godwits are unlikely to be capable of predicting the optimal migratory route before departing on their migration (Dijkstra 1959, but see Gill *et al.* 2014). Despite this caveat, a comparison of the informed and uninformed simulations shows that the differences between experienced and minimal wind assistance are smaller than the larger day–day variation in wind conditions. Our simulation framework should thus be suitable for assessing the *en route* wind conditions of other small migrating bird species that cannot be tracked with GPS transmitters (Bridge *et al.* 2011).

### Trade winds across the Sahara and in‐flight mortality

Although the strength of the trade winds over the Sahara varies between years (Taylor *et al.* 2017), trans‐Saharan migrants seemingly have evolved a diverse suite of strategies to cope with the synoptic phenomena that determine local wind patterns in the region (Kranstauber *et al.* 2015; Evan *et al.* 2016; Vansteelant *et al.* 2017). In the case of godwits, previous studies have shown that they can exhibit flexibility in the timing of their Sahara crossings (Verhoeven *et al.* 2019), dynamically adjust their flight altitude in response to temperature and wind conditions (Senner *et al.* 2018), and have the ability to use emergency stopover sites at the beginning of their southward Sahara crossing (after ~ 500 km) or during the final part of their northward Sahara crossing (after ~ 2000 km). Nevertheless, despite these presumed adaptive behaviours, our results indicate that these are not sufficient to ensure safe crossings of the Sahara, at least not during northward crossings. Not only are godwits likely to perish mid‐crossing when they experience adverse wind conditions but the northward Sahara crossing also has the lowest daily survival rates of any period during the godwit annual cycle (Senner *et al.* 2019). This raises the question: Why have godwits not been able to successfully adjust their migratory flights to cope with low wind assistance? The fact that other migrants have also shown elevated mortality rates during northward crossing of the Sahara suggests that wind conditions during this crossing may be an important bottleneck for trans‐Saharan migrants that do not have the possibility to stop (Klaassen *et al.* 2014; Lok *et al.* 2015).

Why, then, do migrants that are about to cross the Sahara not prepare for the possibility that they encounter poor circumstances, for instance, by increasing their fuel loads? After all, the average extra predicted flight time leading to death is only 6 h. One explanation may be that crossings with a low wind assistance occur too infrequently to lead to the evolution of an adaptive response (Winkler *et al.* 2014). Leaving the question as it stands, our study makes clear that wind assistance is a key factor to the success of long‐distance migration and that it directly impacts the population dynamics of a migratory species (Senner *et al.* 2019). Future work should embrace this new frontier and aim to understand how migrants assess departure and *en route* conditions, and why 6 h of extra flight can be a cause of death in birds that can prepare for over 200 h of non‐stop flight (Gill *et al.* 2009). Part of the answer may have to do with learning, making it likely that migratory behaviours, including the decision to initiate migration, may change over the course of an individual’s life (Sergio *et al.* 2014; Verhoeven *et al.* 2019).

### Wind conditions and global climate change

Increasing global temperatures are projected to cause significant changes to the distribution and viability of the populations of many migratory bird species (Saino *et al.* 2010). However, relatively little attention has been paid to the predicted changes in atmospheric circulations and their potential effects on the viability of migration itself (IPCC & Climate change 2013, but see: Weimerskirch *et al.* 2012, La Sorte & Fink 2017, La Sorte *et al.* 2019). Those investigations that have been undertaken suggest that wind conditions are expected to change as a result of climate change, at least in North and South America (La Sorte & Fink 2017; La Sorte *et al.* 2019), and that the strength of the wind and frequency of storms will increase in the Sahara region (Taylor *et al.* 2017), but the exact effect of these changing wind conditions on trans‐Saharan migrants is largely unknown. Given our findings, which reveal a relationship between experienced wind conditions during migration and the survival of a trans‐Saharan migrant, future studies should aim to understand how changing climatic conditions will affect the wind support of trans‐Saharan migrants and, potentially, their population dynamics.

## Funding Information

This work was supported by the Spinoza Premium 2014 awarded to TP by the Netherlands Organization for Scientific Research (NWO), with critical supplementary funding from the NWO‐TOP grant ‘Shorebirds in Space’ to TP in 2011, an anonymous donor, the Gieskes‐Strijbis Fonds and the University of Groningen – Ubbo Emmius Fonds.

## Authorship

AHJL and MAV conceived the idea and analysed the data; AHJL, MAV, NRS and TP collected the data; AHJL wrote an initial version of the manuscript, which was then substantially edited by all authors.

## Conflict of Interests

The authors declare that they have no competing interests.

## Data Availability

All data needed to evaluate the conclusions in the paper are present in the paper and available from the Figshare Data Repository (https://doi.org/10.6084/mp.fishare.9730733.v1).
