# Supplementary material for: Adverse wind conditions during northward Sahara crossings increase the in‐flight mortality of Black‐tailed Godwits
Source: Ecol Lett. 2019 Sep 17;22(12):2060–6. doi: 10.1111/ele.13387 (PMC6900105; doi:10.1111/ele.13387)
Supplement: Supplementary file 1 [file ELE-22-2060-s001.docx]

**Figure S1. Visualization of the possible connections between the different nodes.** In all three examples, travel starts from the upper middle node. However S1A represents all *horizontal* connections, S1B all *upward* connections to a lower pressure level, S1C all *downward* connections to a higher pressure level. The air speed of the connection in each direction is kept constant at 18.05 m/s^-1^ (Senner *et al.* 2018) and therefore the travel time between nodes is only influenced by the wind speed at a node, as well as the direction and length between two nodes.

Longitude

Latitude

Latitude

Latitude

A)

B)

C)
